# A spatial agent-based model of *Anopheles vagus* for malaria epidemiology: examining the impact of vector control interventions

**DOI:** 10.1186/s12936-017-2075-6

**Published:** 2017-10-27

**Authors:** Md. Zahangir Alam, S. M. Niaz Arifin, Hasan Mohammad Al-Amin, Mohammad Shafiul Alam, M. Sohel Rahman

**Affiliations:** 1 Department of Computer Science & Engineering (CSE), Bangladesh University of Engineering & Technology (BUET), ECE Building, West Palasi, Dhaka, 1205 Bangladesh; 2 Department of Computer Science and Engineering, University of Notre Dame, Notre Dame, Indiana 46556 USA; 3 International Centre for Diarrhoeal Disease Research Bangladesh (icddr,b), Dhaka, 1212 Bangladesh

**Keywords:** Malaria, Agent-based model (ABM), *Anopheles vagus*, Vector control intervention, Integrated vector management (IVM), Larval source management (LSM), Insecticide treated nets (ITNs), Indoor residual spray (IRS), Combined intervention

## Abstract

**Background:**

Malaria, being a mosquito-borne infectious disease, is still one of the most devastating global health issues. The malaria vector *Anopheles vagus* is widely distributed in Asia and a dominant vector in Bandarban, Bangladesh. However, despite its wide distribution, no agent based model (ABM) of *An. vagus* has yet been developed. Additionally, its response to combined vector control interventions has not been examined.

**Methods:**

A spatial ABM, denoted as ABM$_{vagus}$, was designed and implemented based on the biological attributes of *An. vagus* by modifying an established, existing ABM of *Anopheles gambiae*. Environmental factors such as temperature and rainfall were incorporated into ABM$_{vagus}$ using daily weather profiles. Real-life field data of Bandarban were used to generate landscapes which were used in the simulations. ABM$_{vagus}$ was verified and validated using several standard techniques and against real-life field data. Using artificial landscapes, the individual and combined efficacies of existing vector control interventions are modeled, applied, and examined.

**Results:**

Simulated female abundance curves generated by ABM$_{vagus}$ closely follow the patterns observed in the field. Due to the use of daily temperature and rainfall data, ABM$_{vagus}$ was able to generate seasonal patterns for a particular area. When two interventions were applied with parameters set to mid-ranges, ITNs/LLINs with IRS produced better results compared to the other cases. Moreover, any intervention combined with ITNs/LLINs yielded better results. Not surprisingly, three interventions applied in combination generate best results compared to any two interventions applied in combination.

**Conclusions:**

Output of ABM$_{vagus}$ showed high sensitivity to real-life field data of the environmental factors and the landscape of a particular area. Hence, it is recommended to use the model for a given area in connection to its local field data. For applying combined interventions, three interventions altogether are highly recommended whenever possible. It is also suggested that ITNs/LLINs with IRS can be applied when three interventions are not available.

**Electronic supplementary material:**

The online version of this article (doi:10.1186/s12936-017-2075-6) contains supplementary material, which is available to authorized users.

## Background

Malaria is a serious tropical disease spread by mosquitoes. Globally, an estimated 212 million malaria infections occurred during 2015 [1]. It is one of the major health concerns in South-East Asia, including Bangladesh, India, Myanmar, Nepal and Bhutan [2, 3]. The southern part of Bangladesh known as the Chittagong Hill Tracts (CHTs) has been reported as a malaria hotspot and contains some of the highly endemic districts in Bangladesh [4–6]. Some recent studies have reported that 72% of the population are at risk in the east and north-east border belt area of Bangladesh, and the human malaria-transmitting species *Anopheles vagus* is a dominant species in this area, especially in the CHTs [7, 8]. *Anopheles vagus* is also widely distributed in other countries in Asia, particularly in Cambodia, China (including Hong Kong), India, Indonesia, Laos, Malaysia, Mariana Islands, Myanmar, Nepal, Philippines, Sri Lanka, Thailand, and Vietnam [8–10]. However, there is no specific study of *An. vagus* with a focus on controlling malaria transmission in these geographic areas.

*Anopheles vagus* is able to transmit both *Plasmodium falciparum* and *Plasmodium vivax* among humans [7, 11]. Recent studies have reported the vectorial role of *An. vagus* in malaria transmission in many parts of the world [2, 8, 10, 12–18]. Although the species is considered a primary vector in some places, however, due to some recent changes in a variety of global factors (e.g., increasing human populations, global warming, loss of forests and subsequent increases in larval habitats for *An. vagus* etc.), the role of *An. vagus* as a vector of human pathogens has increased significantly. Hence, the need for a detailed model of *An. vagus* including examination of the relative role of the dominant factors on its abundance and its response to vector control interventions are warranted.

**Fig. 1:**
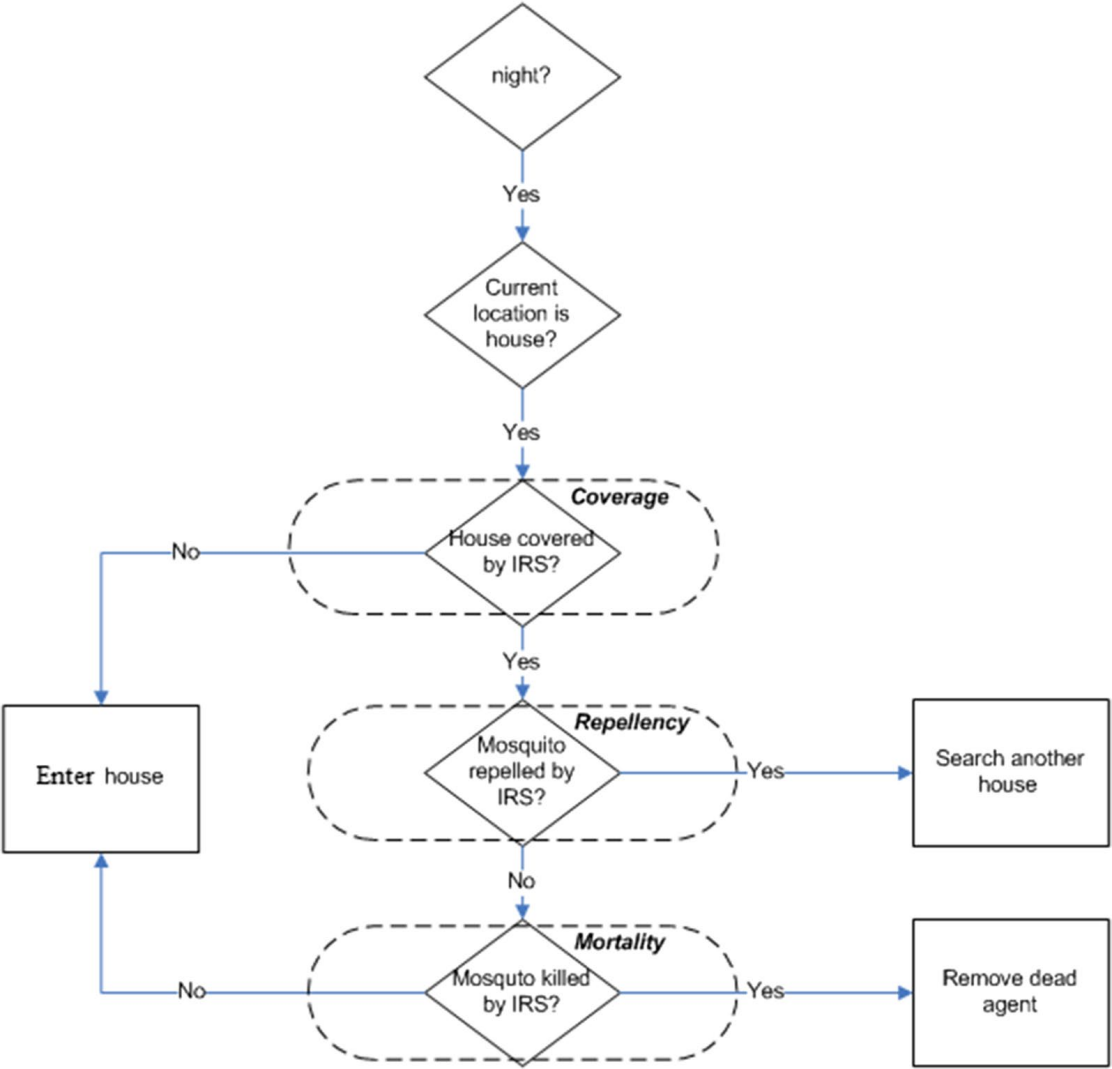
Logical flowchart of IRS intervention. When a mosquito gets a blood meal it has a specific probability to rest within an IRS covered house or get deterred (due to IRS repellency) and move to some adjacent house to rest. If it stays in the IRS protected resting place it may live or die based on the mortality rate of the IRS chemical

**Fig. 2:**
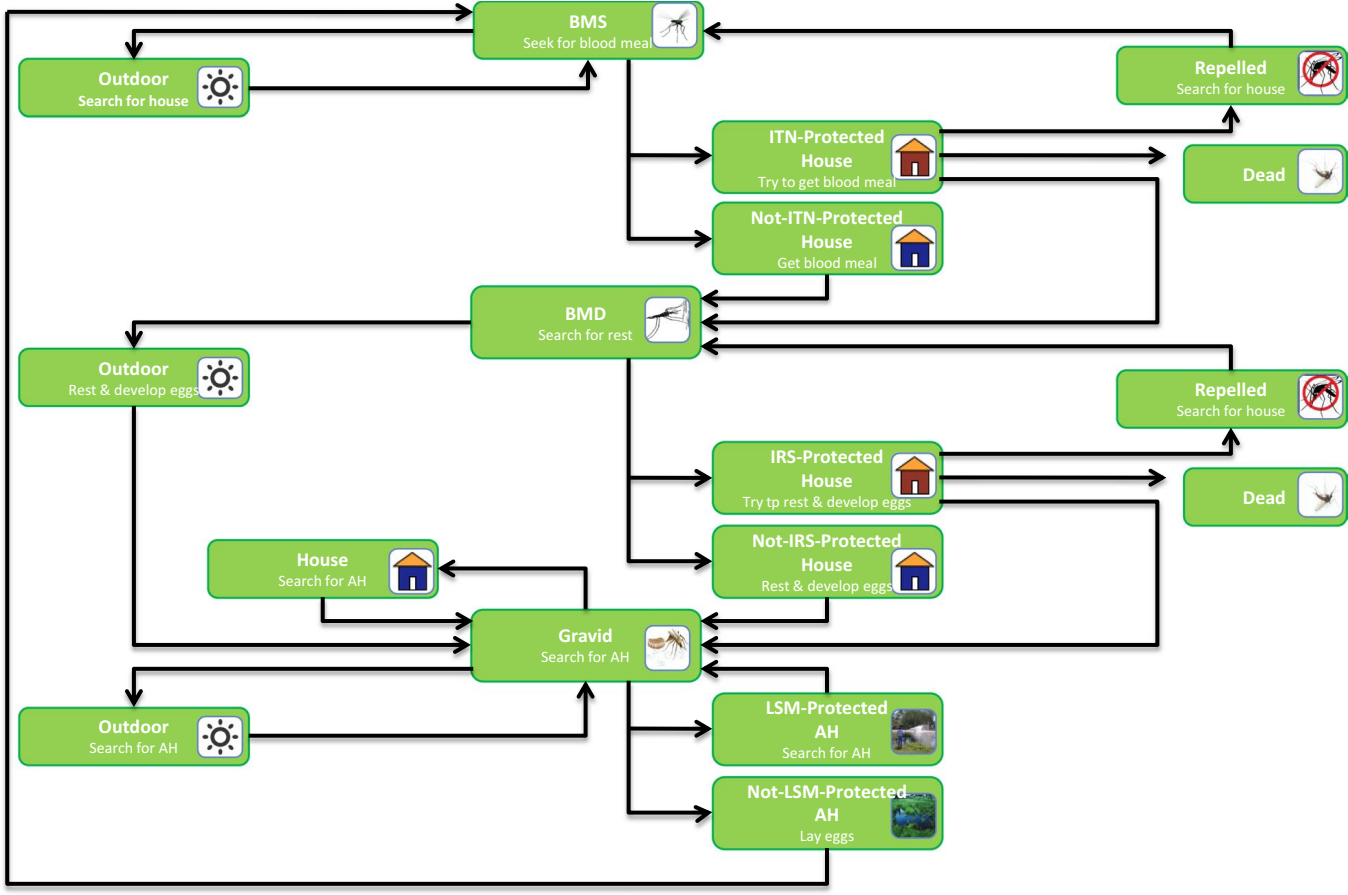
A complete flowchart of applying three interventions in combination. It represents vector’s actions in various states while all interventions are applied in combination. Here, AH means Aquatic Habitat, INT-Protected House means a Household which is protected by ITN, Not-INT-Protect House means a Household which is not protected by ITN, and so on. Every block represents vector’s one of the state with its action in that state

Agent-based models (ABMs), known as the individual-based models, are a type of computational models which have been used to model the basic behaviour of individual mosquitoes and their interactions to the local environment. Several malaria ABMs have successfully addressed different research questions involving malaria [19–30]. However, despite the wide distribution of *An. vagus*, no specific model based on *An. vagus* has yet been developed or reported in the literature.

### Environmental factors

Environmental factors such as temperature, rainfall, and humidity can have major impacts on the vector population dynamics, as indicated by several studies [9, 16, 31–36]. For example, temperature can accelerate the gonotrophic cycle of *Anopheles* mosquitoes, decrease the interval between egg-laying episodes, and increase vector abundance [37–41]. Very high temperature may jeopardize the survival rate of adult mosquitoes and dry up breeding sites more rapidly, resulting lower abundance [42]. For most *Anopheles* species (including *An. vagus*), there is also a significant positive relationship between abundance and rainfall, and the availability of oviposition habitats will increase only if there is enough rain [16]. On the other hand, high rainfall may decrease mosquito abundance by washing out the immature stages [43]. Likewise, other environmental factors such as humidity, cloudiness, and wind speed can also affect malaria transmission [34, 44–46]. Several malaria models have incorporated many of these factors in different ways. For example, Paaijmans et al. [47] have presented a model with the effect of temperature on the larval development. In a very recent study, Arifin et al. [29] have considered temperature as a model input parameter.

The effects of environmental factors on malaria transmission variables are also observed in Bangladesh. For example, Rahman et al. [48] have indicated that incidence is seasonal in Bangladesh where the peak season is identified in the warm and wet months of May–October and the off-peak season is identified in the dry and cooler months November–April. Khan et al. [17] have conducted a study in rural Bandarban (one of the districts of CHTs) and have shown that 85% of the malaria cases are found during the rainy season.

**Fig. 3:**
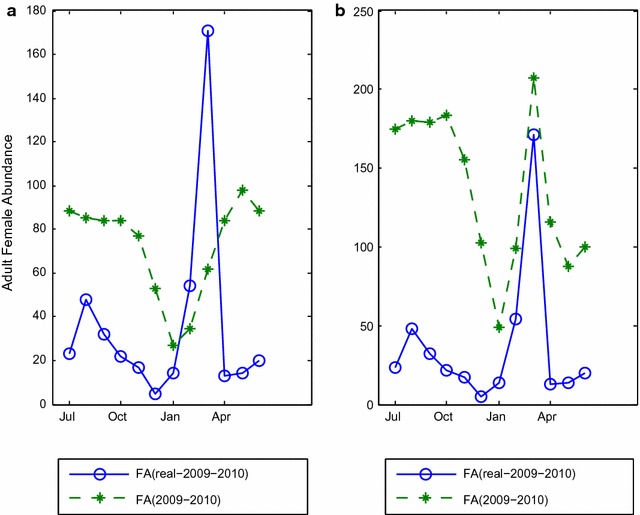
Impact of composite temperatures.** a** Shows the female abundance graph when the daily average temperatures are considered. It shows that the pattern of the curve is not similar with the curve of the real-life data.** b** Shows the female abundance graph when the max temperature for each day in February and March and the min temperature for each day in April and May are considered

It is important to consider daily temperature and rainfall data of a particular area *within* a model to quantify its seasonal patterns of malaria abundance. Some studies (e.g., Arifin et al. [29]) have considered temperature and rainfall as constant input parameters for vector simulations. However, considering the relative importance of temperature and rainfall, ABMs can produce seasonal patterns of vector abundance, yielding, in turn, more interesting and realistic results for a particular period in a specific area.

### Vector control interventions

One of the usages of ABM is to check the performance of some vector control interventions, namely, larval source management (LSM), insecticide-treated nets (ITNs), indoor residual spraying (IRS) etc. These interventions have been extensively used to reduce and control malaria in sub-Saharan Africa [29], Myanmar [49–51], Nepal, Bhutan, Assam state of India [52, 53] etc. Notably, LLINs (long-lasting insecticidal nets), IRS, and LSM are suggested by WHO for South-East Asia [2]. Recent studies have reported that current interventions used/employed in Bangladesh are ITNs/LLINs only [2, 8, 54–56]. Hence, it is important to check the impact of these and other interventions in the context of Bangladesh.

ITNs/LLINs are considered to be one of the key vector control interventions for indoor biting mosquitoes [57–60]. In some literature, ITNs are claimed to be more effective for the indoor mosquito biting when used in conjunction with IRS [19–21, 29, 57, 61–64]. ITNs also provide indirect protection to non bed net users due to insecticides used in the nets [19, 59]. While analysing the impact of ITNs, some earlier models have assumed uniform contact structure between mosquitoes and hosts across the landscape primarily due to mathematical convenience [65, 66]. The proximity between mosquitoes and host can play an important role in the mosquito biting behaviour due to the flight ranges and sensory perceptions of mosquitoes [67–71]. IRS is not used in CHTs, Bangladesh. Protopopoff et al. [72] and Thomsen et al. [73] have identified ITNs and IRS as the two major interventions. Some models have analysed the impact of ITNs and IRS, albeit with some constraints. For example, IRS has been studied without considering its repellency factor in a discrete-space continuous-time mathematical model in Lutambi et al. [74, 75] and in a malaria transmission-directed model in Eckhoff’s research in 2011 [21]. For applying IRS in spatial ABM requires to consider coverage, repellency and mortality factors. Hence, the ITN/LLIN as well as IRS need to be studied and analysed in a spatial based model.

**Fig. 4:**
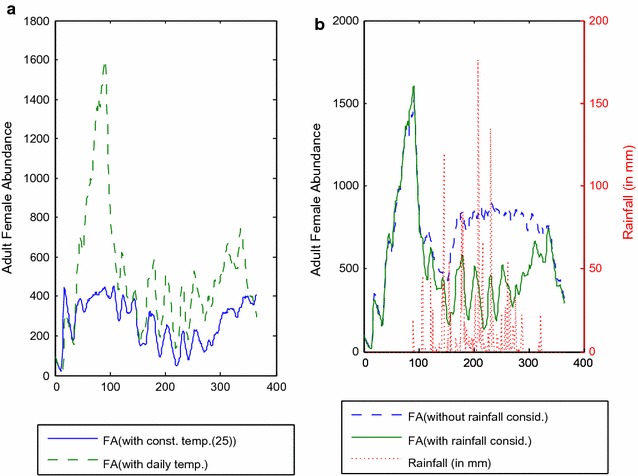
Impact of environmental factors.** a** Shows the comparison of female abundance of a simulation between constant temperature (e.g., 25 °C) usage and the daily temperature usage from the weather profile data for the full simulation period. Two curves present two different types of output.** b** Shows the comparison of female abundance of a simulation between the output of no rainfall/constant rainfall data (i.e., 1.0) usage (referred to as NR) model and the daily rainfall data usage (referred to as DR) model where the later assumes the daily rainfall data from a weather profile. The differences are very clear in the figure

Another useful intervention is LSM. Fillinger and Lindsay [76] have reported LSM as a successful method in Africa by highlighting historical and recent successes. The effectiveness of LSM of an area depends on the understanding of hydrology and geomorphological process which governs the availability and productivity of *Anopheles* breeding habitats at a local scale [45]. Although LSM sometimes are not used due to high cost, it is cost effective compared to ITNs and IRS in areas where aquatic source are accessible and well defined [77]. LSM is not still used in Bandarban. Hence, its performance needs to be measured on the landscape of Bandarban.

Another important dimension is the combination of interventions. Some researchers [76, 78–80] have discussed the potentiality and success of integrated vector management (IVM) approach. For instance, the reduction of prevalence of malaria in Botswana was deduced to be approximately 98% within five years (2008–2012) while LLINs and IRS are used in combination [81]. Although ITNs reduced malaria mortality and morbidity in Africa consistently, their benefits are less consistent in Asia [82]. This can be attributed to the fact that vector mainly bites in the evening, often before people are protected by ITNs as observed in western Myanmar [82]. Additionally, increasing concerns of resistance of mosquitoes to insecticides are reported in the literature[72, 73, 83–88]. All these suggest to think about alternative interventions (e.g., LSM) along with chemical interventions (e.g., ITNs, IRS).

LLINs are believed to be less effective in areas with pyrethroid resistant vectors [87]. Some insecticides which are used in ITNs/LLINs and IRS have less toxicity and hence are less effective against mosquitoes. Thus, LSM can play crucial role in combination.

**Fig. 5:**
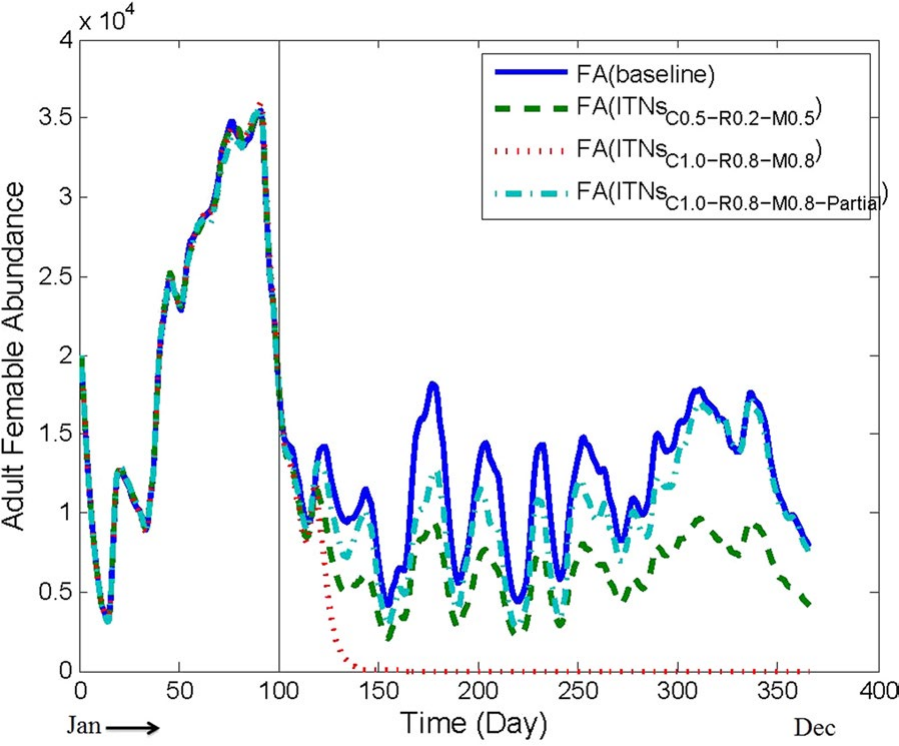
Comparing baseline abundance with ITNs being applied in isolation with different parameter values from Table 4. The X-axis presents the day of a year and the Y-axis presents the FA for a particular day. For the parameter setting #1 in Table 4, with *household-level complete* scheme, the graph clearly exhibits significant reduction of the FA. For the parameter setting #2 in Table 4 with *household-level complete* scheme, the FA decreases drastically after applying ITNs; however, with *household-leve partial* scheme, the FA decreases in a lower rate

This paper makes the following key contributions.

- The design and implementation of an ABM for *An. vagus* (referred to as ABM$_{vagus}$ henceforth) are described based on the life cycle of *An. vagus*. This ABM is designed incorporating the biological phenomena of *An. vagus* reported in the literature, real-life field data on them and mathematical equations found for the generic *Anopheles* species in the literature. The logics are designed and implemented for incorporating some environmental factors more accurately than the other works in the literature.
- The verification and validation (V and V) of ABM are performed using docking techniques and with real life field data.
- The impact of the environmental factors over the output of ABM is examined and the seasonal pattern of vector abundance is presented for a particular area.
- The logic of applying IRS into ABM as well as the existing implementation logics of some other interventions (e.g., ITNs and LSM) are incorporated and implemented in ABM$_{vagus}$. The impact of vector control interventions (e.g., ITNs, IRS, LSM) over vector population dynamics by ABM is examined to quantify the performance of the interventions while they are used in isolation mode as well as in combination.

The ABM presented in this paper (denoted as ABM$_{vagus}$) has been developed using Java [89, 90] by modifying an established existing ABM for *An. gambiae* (referred to as ABM$_{gambiae}$ henceforth) [26–29]: a brief comparison between the two based on the differential features only is shown in Table 1. The code and relevant data for ABM$_{vagus}$ is provided in Additional files 2, 3, 4.

**Table 1:** Major differential feature comparison between ABM$_{gambiae}$ and ABM$_{vagus}$

Model features	ABM$_{gambiae}$	ABM$_{vagus}$
Mosquito Species	*Anopheles gambiae*	*Anopheles vagus*
Model of the egg stage	Basically equation based	Based on field data (probability based)
Model of the pupal stage	Basically equation based	Based on field data (Probability based)
Daily Temperature incorporation	Temperature is variable but constant through full simulation run	Daily temperature is used from a weather profile
Daily rainfall data incorporation	Rainfall coefficient is constant (i.e., 1.0)	Daily rainfall is used from a weather profile
Modification of daily mortality rate (DMR) of egg for Rainfall	No	Yes
Modification of DMR of larvae for rainfall	No	Yes
Modification of DMR of pupae for rainfall	No	Yes
Seasonal pattern of vector abundance	No	Yes
Landscapes	Generated by *VectorLand*	Generated by *VectorLand* and landscapes of Bandarban
Individual interventions modeled	ITNs, LSM	ITNs, IRS, and LSM
Versions	–	A number of versions of the model considering different parameter combinations as well as based on biological life cycle (e.g., 8 vs. 12 stages) have been implemented

## Methods

Several life-cycles, namely, the malaria transmission cycle, life-cycle of the mosquito, and life-cycle of the malaria parasite, are normally considered for modelling malaria. Following the work of Zhou et al. [26] and Arifin et al. [29], this study only models the life-cycle of the mosquito. In the following subsections, we describe the development of ABM$_{vagus}$ in detail.

### Model development

ABM$_{vagus}$ has been developed by modifying ABM$_{gambiae}$. The goal of ABM$_{vagus}$ is to simulate the core model of the vector *An. vagus*. ABM$_{vagus}$ holds the state transitional logic according to the life-cycle of *An. vagus* based on the attributes and environmental information.

**Fig. 6:**
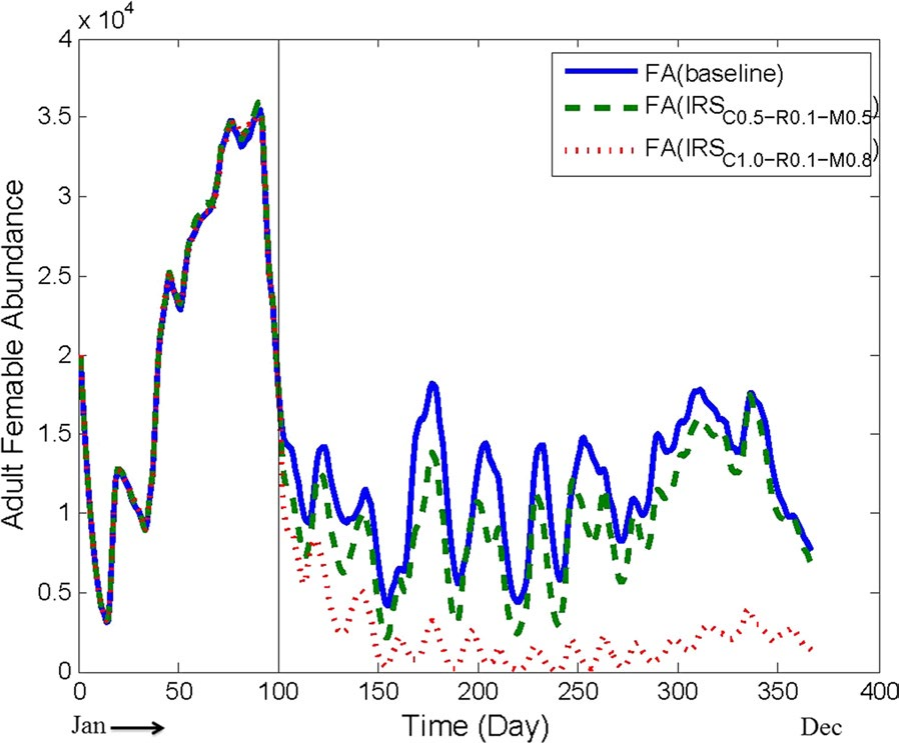
Comparing baseline abundance with IRS being applied in isolation with different parameter values. The X-axis presents the day of a year and the Y-axis presents the FA for a particular day. For parameter setting #3 in Table 4, after applying IRS, FA is decreased albeit insignificantly. For parameter setting #4 in Table 4, the FA is decreased significantly

Like all other *Anopheles* mosquitoes, *An. vagus* also goes through four main stages in their life cycles, namely, egg, larva, pupa, and adult. The first three immature aquatic stages are broadly the same in all mosquitoes. Hence, the following distinct aquatic stages are considered into the core model: *Egg (E)*, *Larva (L)* and *Pupa (P)*. During adult stage, the process of blood feeding, egg development inside female mosquito and oviposition are repeated several times throughout the life-cycle until the female dies. Following the recent work of Zhou et al. [26] we therefore have further included the following stages in ABM$_{vagus}$: *Immature Adult* (*IA*), *Mate Seeking* (*MS*), *Blood meal seeking* (*BMS*), *Blood meal digesting* (*BMD*), and *Gravid* (*G*).

#### The egg stage

Mohammad Shafiul Alam and Hasan Mohammad Al-Amin has stated that 60% eggs of *An. vagus* are developed within 2 days, and the remaining 40% within 3 days in normal temperature (e.g., 26–30 °C) (personal communication, January 15, 2014). A probability model was developed based on the above and adopted into the core model for the egg stage.

**Fig. 7:**
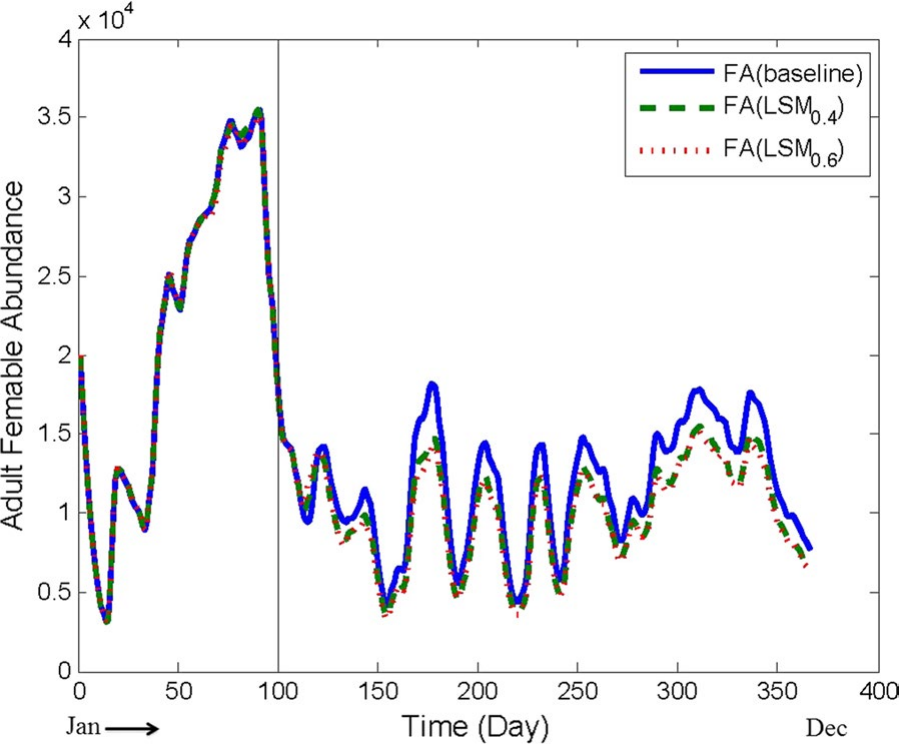
Comparing baseline abundance with LSM being applied in isolation with different values of coverage. The reduction in FA is not significant. However, the FA reduction is consistent throughout the year and for higher coverage the reduction is higher

**Fig. 8:**
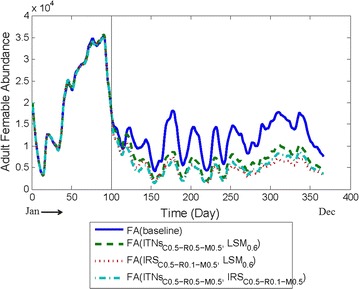
Comparing baseline abundance with two interventions being applied in combination

#### The larval stage

Since temperature is a critical variable in the growth and development kinetics of *Anopheles* mosquitoes, a temperature driven framework [26, 29] is directly incorporated into the core model. For the sake of brevity we omit the details of this framework [26, 29] here. The readers are kindly referred to Additional file 1 for more details.

#### The pupal stage

Based on the unpublished laboratory data provided by two co-authors, HMA and MSA, 40% of pupae are developed within 24 h and the rest 60% of pupae are developed within 30 h. This probability based model is adopted into the core model for the pupal stage.

#### The immature adult stage

As the temperature determines the duration of this stage, a temperature-dependent model [30] has been adopted for this stage into the core model. Details of the temperature-dependent model [30] are given below in Additional file 1.

#### The mate seeking stage

For this stage, the model of Arifin et al. [29] (Please refer to Additional file 1 for details) has been incorporated into the core model.

#### The blood meal seeking stage

The different spatial factors, e.g., location, resource density, the movement of the mosquito, landscapes etc., along with the incorporation logics mentioned in Arifin et al. [29] are incorporated into the core model as the logics are very generic for *Anopheles* mosquitoes. Furthermore, the most active time period of *An. vagus* has been identified and considered during the complete 24-h period in a day as follows. Nagpal and Sharma reported in 1995 that the biting time of female *An. vagus* is before midnight with a peak time between 18.00 and 20.00 h [91]. However, in 1963 Quraishi [92] rigorously examined each time period to check the activities of *An. vagus* in Bangladesh and identified the peak time to be 5.00 and 6.00 h (i.e., in the morning) (Table 2). The contradiction between the findings of the two works mentioned above had left us no choice but to select only one of the two in the core model. The report of Quraishi in 1963 [92] contains the relevant data regarding *An. vagus* in detail and it is conducted in Bangladesh and we have the real-life data of female abundance of Bandarban, Bangladesh. Additionally *An. vagus* are observed in the morning resting indoor with relatively fresh blood which indicates their biting during very early morning during field data collection period (Al-Amin, HM and Alam, MS, personal communication, 2013). Thus, the data of [92] have been adopted into the core model.

**Table 2:** Nocturnal activities of *An. vagus* in different time period from 8.00 p.m. to 8.00 a.m. based on [92]

Time period	Activity in percentage
8.00 p.m. to 9.00 p.m.	0
9.00 p.m. to 10.00 p.m.	13.67
10.00 p.m. to 11.00 p.m.	15.83
11.00 p.m. to 12.00 a.m.	11.30
12.00 a.m. to 1.00 a.m.	7.2
1.00 a.m. to 2.00 a.m.	0.72
2.00 a.m. to 3.00 a.m.	0
3.00 a.m. to 4.00 a.m.	0.72
4.00 a.m. to 5.00 a.m.	1.44
5.00 a.m. to 6.00 a.m.	*35.25*
6.00 a.m. to 7.00 a.m.	14.39
7.00 a.m. to 8.00 a.m.	0

#### The blood meal digesting stage

As this stage is usually highly temperature dependent, the temperature-dependent linear function developed in other model [30] was adopted into the core model. More details on this model are given below in Additional file 1.

#### The gravid stage

The (generic) ovipoisitioning rules and models for gravid stage presented in Zhou et al. [26], Arifin et al. [29] and Arifin et al. [30] is adopted into the core model. More details on this model are given below in Additional file 1.

#### Mortality in the adult stages

For this stage, the mortality model used in Zhou et al. [26] and Arifin et al. [30] has been adopted. This model can be seen as a modified version of the logistic mortality model in which the age-dependent component of mortality increases exponentially with age. For details please refer to Additional file 1.

#### Mortality in the immature stages

Since immature mosquitoes (i.e., Egg, Larva, Pupa) live in the aquatic habitats with variable environmental factors affecting the stages, their mortality rates are considered separately for each stage. According to the field data on *An. vagus* egg (Al-Amin, HM and Alam, MS, personal communication, 2013), 10% eggs die during hatching in aquatic environment ($DMR_{b} (E)$) and 5% of the *An. vagus pupae* die during the pupal period ($DMR_{b}(P)$). Along with the above, an age specific mortality rate for the larval stage ($DMR_{a}(L)$) has been developed recently in [26, 29, 30]. All these are incorporated in the core model with some modifications to incorporate the effect of temperature and rainfall as discussed in the following sections.

**Fig. 9:**
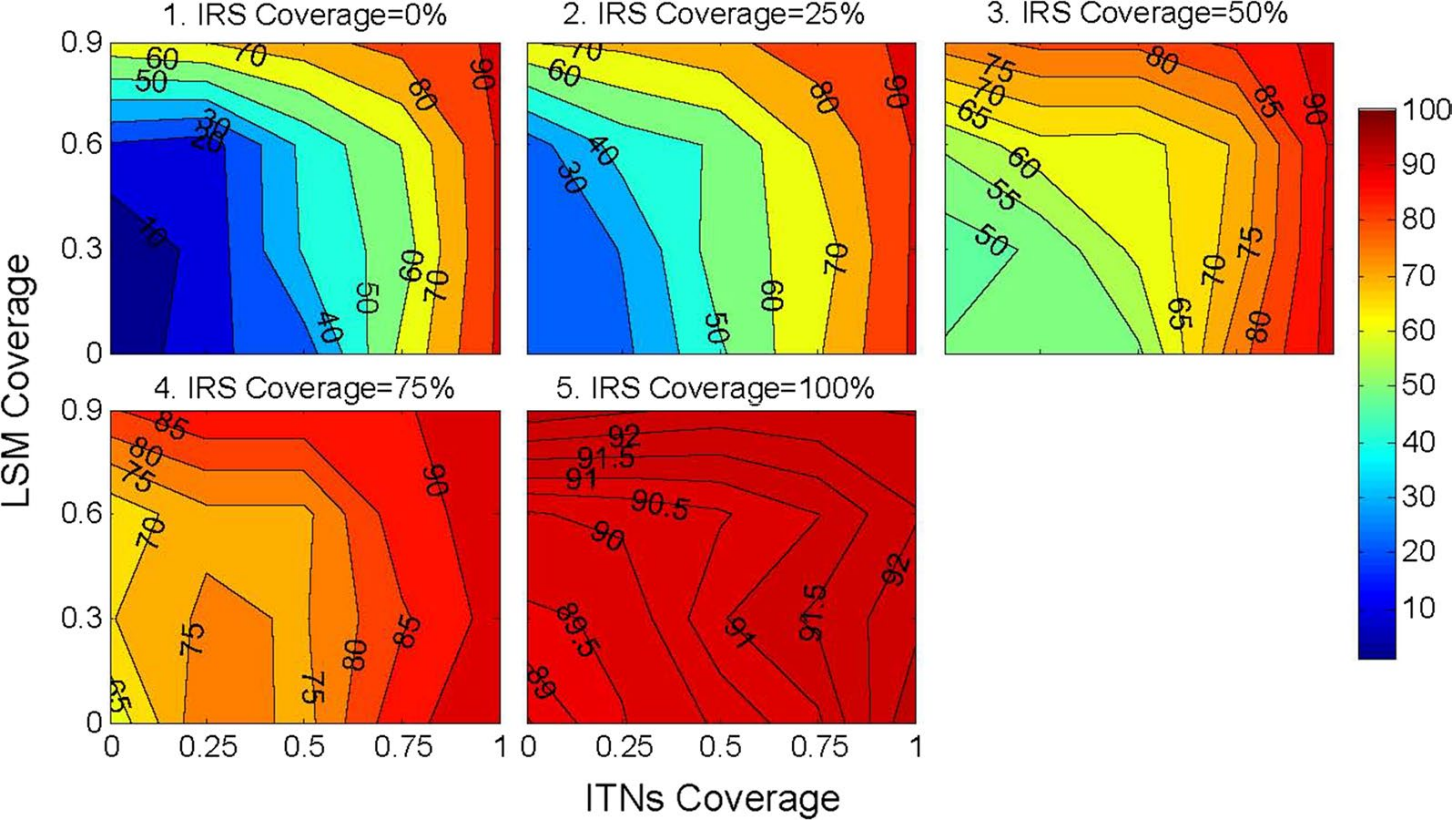
This figure shows the percent reductions in mosquito abundance as a function of LSM coverage, ITNs coverage and IRS coverage when LSM, ITNs and IRS are applied in combination. The X-axis denotes ITNs coverage and the Y-axis denotes LSM coverage. Each subfigure represents FA reduction percentage for LSM and ITNs coverage with fixed a IRS coverage. ITN and IRS mortality (M) are fixed at 0.5, ITN repellence (R) is fixed at 0.5 and IRS repellence (R) is fixed at 0.1. Each simulation is run for 1 year; LSM, ITNs and IRS are applied at day 100, and continued up to the end of the simulation. The filled contour plots in each subfigure shows isolines which are labelled with specific percent reduction (PR) values. The colourbar at the right side quantifies the PR isolines. The figure represents average percent reduction values of FA of a total of 500 (4 × 5 × 5 × 5) simulations. For ITNs, household-level complete coverage scheme with non-absorbing boundary is used

#### Incorporating daily temperature

Water temperature mostly affects the aquatic stages and air temperature mostly affects the adult stage. A *weather profile* has been used in various stages in the mosquito life cycle in the core model. The weather profile keeps daily record of air temperature. The *weather profile* has the maximum and minimum temperature for each day of the year during July 2009–Jun 2010. The daily average temperature, stored in the weather profile, is naturally calculated by taking the average of the above-mentioned two temperatures for each day. Note that in the implementation and simulation the maximum temperatures recorded for each day in February and March, and the minimum temperatures recorded for each day in April and May and the average temperatures for each day in other months have been used.

At this point, a brief discussion on the rationale behind using such a composite temperature profile is in order. In March in Bangladesh, most part of a day usually exhibit a temperature which is nearer to the maximum temperature of the day; but sometimes the minimum temperature may go quite low albeit it stays there for insignificant amount of time. Now, averaging the temperature gives a completely wrong measure of the actual temperature of the day! This and similar argument for some other months led us to adopt the above-mentioned composite temperature profile. Notably, the model is configurable to pick either maximum or minimum or average for each month.

#### Incorporating daily rainfall

Rainfall has major effect on the mortality of larvae, pupae, and eggs. In following sections the modified daily mortality is described in detail.

#### Rainfall consideration for larval mortality

Several studies [16, 93–96] have mentioned the relationship of rainfall and larval decrease. Hence, this is considered into the model of the larval mortality rate. Some studies [26–29] have considered a rainfall co-efficient in the larval age specific mortality rate. But the model [26–29] has assumed rainfall co-efficient as constant (i.e., 1.0). Generally, daily rainfall data is collected in mm form. If daily rainfall data is directly applied into the equation of DMR for larva then the result produced by the equation contradicts some results derived from real life data [43]. To avoid this apparent conflict and contradiction, the equation is modified. The modified equation is described below.

Parham et al. [44] have developed a mathematical model for assessing the effects of rainfall, cloudiness, wind speed, desiccation, temperature, relative humidity and density-dependence on vector abundance [44], where daily survival probability of immature stage exponentially decrease with rainfall increase. By considering rainfall-induced mortality, survivorship is modelled as follows:

1 $$\begin{aligned} \rho _s(R_t)~=~ e^{(-\sigma _s(R_t))} \end{aligned}$$

Here, $\rho _s(R_t)$ represents the daily survival probability of immatures in stage *s* for the given rainfall $R_t$ (in mm.) of the $t^{th}$ day. And $\sigma _s$ quantifies the decrease in survival of stage *s*. By taking $\sigma$ as a input variable of an environment the mortality rate of larvae has been modelled by combining it with the daily mortality rate of larvae. Using Eq. 1 the daily mortality rate of larvae due to rainfall is defined as follows:

2 $$\begin{aligned} DMR_{r}(L) = 1 - \rho _{L}(R_t) \end{aligned}$$

The mortality equation of larva based on the age-cohort model defined by Arifin et al. [29], termed as $DMR_{a}(L)$, and the Eq. 2 based on the effect of rainfall, termed as $DMR_{r}(L)$, are not mutually exclusive. Considering probability theory for not mutually exclusive events, the mortality rate for larvae in total is calculated as follows:

3 $$\begin{aligned} DMR_{t}(L) =~P(DMR_{a}(L)~\mathrm {or}~DMR_{r}(L)) \end{aligned}$$

Applying probability theory, the following equation is generated:

4 $$\begin{aligned} DMR_{t}(L) & = P(DMR_{a}(L))+P(DMR_{r}(L)) \nonumber \\ & \quad-P(DMR_{a}(L)~\mathrm {and}~DMR_{r}(L)) \end{aligned}$$

Following equation is generated considering independent events:

5 $$\begin{aligned} DMR_{t}(L) &= P(DMR_{a}(L))+P(DMR_{r}(L)) \nonumber \\ & \quad-P(DMR_{a}(L)) \times P(DMR_{r}(L)) \end{aligned}$$

The above equation can be written in simple form as:

6 $$\begin{aligned} DMR_{t}(L) &=DMR_{a}(L)+DMR_{r}(L) \nonumber \\ & \quad-DMR_{a}(L) \times DMR_{r}(L) \end{aligned}$$

Equation 6 is used into the core model.

#### Rainfall consideration for egg mortality

The daily mortality rate for eggs increases due to rainfall [44]. By considering rainfall-induced mortality for eggs, using Eq. 1 for the egg stage and the approach mentioned for the larval stage above, the daily mortality rate of eggs due to rainfall is defined as follows:

7 $$\begin{aligned} DMR_{t}(E) & =DMR_{b}(E)+DMR_{r}(E) \nonumber \\ & \quad - \,DMR_{b}(E) \times DMR_{r}(E) \end{aligned}$$

Equation 7 is used into the core model.

#### Rainfall consideration for pupal mortality

The daily mortality rate for pupa also increases due to rainfall [44]. By considering rainfall-induced mortality for pupae, using Eq. 1 for the pupal stage and the approach mentioned for the larval stage above, the daily mortality rate of pupae due to rainfall is defined as follows:

8 $$\begin{aligned} DMR_{t}(P)&=DMR_{b}(P)+DMR_{r}(P) \nonumber \\ &\quad-\,DMR_{b}(P) \times DMR_{r}(P) \end{aligned}$$

Equation 8 is used into the core model.

#### Output indices

Following some recent reports [29, 30], adult female abundance is the primary output of the core model. The abundance of egg, larva and pupa will also available as the secondary output.

#### Model assumptions

ABM$_{vagus}$ is based on mixed combination of theoretical models/equations and field-based data. In the core model a few assumptions have been made. In some cases, these assumptions have been made following the recent works in the literature and hence the same limitations suffered by those works have been inherited; in a few cases however, this research work has recovered from the limitations of previous works. These assumptions are highlighted below.

- Only the life cycle of mosquitoes is considered rather than full malaria transmission cycle that also includes the life cycle of parasites. The model does not separately consider the malaria incidence or malaria infected mosquitoes or malaria infected human.
- The probability of death of a mosquito which increases with age, is used like other models [26, 30].
- The human population is assumed to be static like other models in the literature [26, 30]. All humans are treated to be identical. Other alternative hosts for blood (e.g., cattle, animal) are not considered.
- Only temperature and rainfall are considered as the environmental factors in the core model like others [26, 30]. However, unlike Zhou et al. [26], Arifin et al. [30] who have used temperature as a constant input for the simulation period, in the core model daily temperature is supplied from a weather profile. Hence, the model is able to produce the seasonal pattern of vector abundance which is not possible in prior models.
- Daily rainfall data is applied into the mortality rate of each immature stage. Hence the limitation of Zhou et al. [26] and Arifin et al. [30] is that the rainfall coefficient is set as 1.0 in the daily mortality rate for larvae, has been overcome. The modified equations of these mortality rates use the decreasing quantifier in the survival of the egg, larval and pupal stages as 0.0242, 0.0127, and 0.00618, respectively. These values are collected from Parham et al. [44]. However, the effect of heavy rainfall on the increment of habitats is not considered.
- In several cases theoretical approaches of other *Anopheles* species are directly applied due to unavailability of actual data of *An. vagus*.
- In the *MS* stage, a female is assumed to always find a male mosquito to mate. Also, a single blood meal is assumed sufficient for the maturation of egg. The mortality rate of female adults is treated as independent of their malaria infectivity states. The fecundity of female adult is assumed normally distributed with a mean of 170 and standard deviation of 30.
- Time step in the simulation is modeled on an hourly basis (instead of daily) which provides better granularity than the other works in the literature.
- Two types of grid-based landscapes have been considered in the simulation. The generic one is created by *VectorLand* tool [29]. The other one is generated using some custom conversion based on some field data of Bandarban (Al-Amin, HM and Alam, MS, personal communication, 2013). The former has used fixed carrying capacity (CC) of 1000 each and the latter has used varying capacity based on field data. Each landscape is of size $40 \times 40$ where each cell area is $50\;m \times 50\;m$. How many numbers of breeding sources may be required to fulfill a cell of $50\;m \times 50\;m$ are also assumed.

#### Simulations

The landscape information, daily weather profile with the temperature and rainfall data are given as input in the simulation runs. Initially, 20000 female adult gravid mosquitoes are assumed which are able to lay eggs with 50:50 male–female ratio. Each simulation is repeated at least five times to eliminate any bias introduced by different sources of randomness (stochasticity), the behaviour uncertainties of the agents (i.e., vectors) actions, states, etc. Although daily weather profile data for 4 years for Bandarban are available, the simulation has been run for 1 year for which the real-life data of Female Abundance are available. On the other hand, the runtime of a sample simulation is about 5 h when the landscape generated by *VectorLand* tool is used. A machine with Intel (R) Core (TM) i3 CPU and 8 GB RAM has been used for the simulations; each simulation has been run as a single-threaded program, in a single-process.

### Field data

Three types of field data have been collected as follows. The first one is *An. vagus* abundance [8], the second one is related to stage duration and mortality rate of *An. vagus* (Mohammad Shafiul Alam, personal communication, January 15, 2014 and Hasan Mohammad Al-Amin, personal communication, January 15, 2014) and the third one is landscape related data for Bandarban. Field data on *An. vagus* abundance are collected from Alam et al. [8] where the abundance data of several local species are reported as follows: *Anopheles jeyporiensis*: 18.9%, *An. vagus*: 16.8%, and *Anopheles kochi*: 14.4% of the total 2576 collected *Anopheles* mosquitoes. Monthly *An. vagus* female abundance is reported to reach the highest level during March, followed by an immediate sharp decrease during April. In Fig. 3, monthly *An. vagus* abundance for a year for the study area is shown. According to [8], CDC miniature light traps were used for trapping mosquitoes during their field study. Traps were placed for 12 h (6 p.m. to 6 a.m.). During the wet and dry seasons, 100 and 50 houses respectively were selected randomly for the study. Thus, it is fair to assume that more traps in many more houses would resulted in a much higher figures in abundance data.

Weather data reported in Alam et al. [8] from the Soil Resource Development Institute for Bandarban [97] have been used for the study period. The maximum (max) and minimum (min) temperature values are available in the weather profile. Weather data is collected for four consecutive years (i.e., 2009–2012).

The information on households and the aquatic habitats with their densities and patterns are required for a particular area to create the corresponding landscape for ABMs. Rice fields, animal hoof prints, large artificial containers, bamboo holes, and Puddles are major oviposition habitats (known as breeding sources) for *An. vagus* in the rural area of Bandarban [98]. Table 3 presents the ratio between households and breeding sources that have been used to generate the landscapes.

**Table 3:** Household ratio with breeding sources in general

Ratio items	Ratio in general
Rice field:household	0.5:1
Animal hoof print:household	2:1
Large artificial container:household	1:1
Bamboo hole:household	2:1
Puddle:household	1:1

The other data related to the landscape are generated based on the study of Khan et al. [17]. The authors of this study surveyed two unions, namely, Rajbila and Kuhalong, that cover 179 square kilometres at CHTs having a total of 5050 houses (2320 in Rajbila and 2730 in Kuhalong). Thus, houses per square kilometre are calculated to be 28.21. Hence, a landscape of $40 \times 40$ cells with each cell occupying $50\;m \times 50\;m$ area can be prepared by placing a total of 112.85 houses in a 4 square kilometres area. Each breeding source is assigned with a CC number per unit. The number of breeding sources required to fulfill a cell of $50\;m \times 50\;m$ is assumed. Using these two information along with above mentioned ratio the total CC is calculated for a cell for a particular breeding source type. For example, household ratio with puddle is 1:10, one puddle has carrying capacity 15, 125 puddles are required to fulfill a $50\;m \times 50\;m$ area. Hence, the total CC per cell is calculated as $125 \times 15= 1875.$ For a $40 \times 40$ landscape the total number of puddles is $112.85\times 10= 1128.50.$ These puddles can cover $1128.50 / 125 = 9.028$ cells. So, there will be 9 cells of puddle each having a CC of 1875. To account for the fractional (0.028) part, an additional cell with a CC of $0.028 \times 1875=52.5$ is created.

### Vector control interventions

Based on the practical usages of various interventions along with recommendations in the literature, ITNs, IRS, LSM are considered for examining their effect on the vector population dynamics. The interventions are applied alone or in combination. The logic for ITNs and LSM as developed in Arifin et al. [29] is directly incorporated into our model. Additionally, we have developed a logic for IRS intervention which is discussed subsequently.

#### IRS modelling

Generally, IRS works when mosquitoes rest on the sprayed area after getting blood meals. Identifying the resting places of mosquitoes accurately within a house is difficult. Similarly, accurately measuring how much area has been sprayed is also difficult. Hence, for simplicity, it is assumed that if a house is completely covered by IRS then all resting places (like walls, ceiling, roof etc.) are well sprayed. When a mosquito gets a blood meal it has a specific probability to rest within a IRS covered house or get deterred (due to IRS repellency) and move to some adjacent house to rest. If the female mosquito stays in the IRS protected resting place it may live or die based on the mortality rate of the IRS chemical. A simple flowchart in Fig. 1 captures this logic.

There are three IRS parameters, namely, coverage (C), repellency (R) and mortality (M) that are defined by probability distribution functions. For example, an IRS coverage of 0.4, repellency factor of 0.25 and mortality factor of 0.20 indicate that 40% of the total houses are covered by IRS, 25% mosquitoes are repelled and 20% mosquitoes are killed when they try to take rest on the surfaces of a IRS covered house, respectively.

#### A complete flowchart of applying interventions in combination

Figure 2 presents a complete flowchart for applying all interventions in combination.

#### Applying ITNs in isolation

To study the response of host-seeking mosquitoes to ITNs, a strategy to apply ITNs is modelled based on three parameters, namely, *coverage C, repellence R,* and *mortality M*. In the simulation, ITNs intervention is applied when a female mosquito is in the *Blood meal seeking* stage. Note that in [29], three different schemes were designed and docked with previous results. These schemes were household-level partial coverage with single chance, household-level partial coverage with multiple chances and household-level complete coverage. It is important to check the impact of these three schemes when applied into the landscapes of Bandarban. All three schemes are input variables for the model. Based on the configuration only one scheme at a time is used during the simulation. For this experiment, 40 × 40 sized landscape of Bandarban has been used with the parameter values for ITNs shown in Table 4.

**Table 4:** Parameters for applying interventions in isolation and in combination

No.	Combination	Coverage	Repellence	Mortality
1	ITNs in isolation	0.5	0.2	0.5
2	ITNs in isolation	1.0	0.8	0.8
3	IRS in isolation	0.5	0.1	0.5
4	IRS in isolation	1.0	0.1	0.8
5	LSM in isolation	0.4		
6	LSM in isolation	0.6		
7	ITNs and LSM	0.5, 0.6	0.5	0.5
8	IRS and LSM	0.5, 0.6	0.1	0.5
9	ITNs and IRS	0.5, 0.5	0.5, 0.1	0.5, 0.5

**Table 5:** Parameters for applying three interventions in combination

Combination	Coverage(s)	Repellence	Mortality
LSM	0.0, 0.3, 0.6, 0.9	0.5	0.5
ITNs	0.0, 0.25, 0.5, 0.75, 1.0	0.5	0.5
IRS	0.0, 0.25, 0.5, 0.75, 1.0	0.1	0.5

#### Applying IRS in isolation

IRS application is also modelled on the above-mentioned three parameters used for ITNs. In the simulation, IRS intervention is applied when a female mosquito is in the *Blood meal digesting* stage. Here as well, 40 × 40 sized landscape of Bandarban has been used for this experiment using the parameter values for IRS shown in Table 4.

#### Applying LSM in isolation

The impact of LSM in isolation has been investigated following the strategy of [29] as follows. Some of the aquatic habitats in the 40 × 40 sized landscape generated for Bandarban are marked as invalid (based on the LSM coverage percentage). The input parameters with the landscape also contain the time of LSM application. During those pre-specified times, all invalid marked aquatic habitats are removed from the landscape and as a result no mosquito can lay eggs on those. The parameter values for LSM shown in Table 4 are used in this experiment.

#### Applying two interventions in combination

Several combinations of vector control interventions have been studied and investigated, namely, ITNs with IRS, ITNs with LSM, and IRS with LSM. When ITN is combined with IRS, ITN repellency is assumed to be fixed at 0.5 and other two factors (C and M) are varied. The parameter values for two interventions in combination shown in Table 4 are used in this experiment. The 40 × 40 sized landscape of Bandarban has been used for this experiment.

#### Applying ITNs, IRS, and LSM in combination

ITNs, IRS, and LSM in combination have been applied using parameter values shown in Table 4. The 40 × 40 sized landscape of Bandarban has been used in this experiment.

#### Assumptions for interventions

Following the relevant literature, some assumptions have been made. For ITNs, following [29], transient effects, such as, the decay of insecticide effectiveness of the bed nets, are ignored. Furthermore, the complete usage (adherence) has been assumed. In other words, humans provided with a bed net are always assumed to sleep under it during night. This study also assumes all ITNs/IRS parameters (C, R, and M) to be constant over time, and ignore any possible development of insecticide resistance in the mosquitoes. For IRS, it is further considered that all places are sprayed completely and uniformly. For LSM, like other study [29], all aquatic habitats are assumed to have no inherent differences in their attractiveness and productivity. Notably, when no intervention is applied into the model, the mosquito population is governed by the combined carrying capacities of all aquatic habitats, and the density-dependent oviposition mechanism; the latter limits the potential number of eggs that a female mosquito may preferentially lay in an aquatic habitat, considering both the associated CC and the *biomass* already present in the habitat.

#### Simulations

Application of interventions to the model is parameterized and taken from input. Intervention(s) are applied after 100 days, and continued up to the end of the simulation. This is to ensure that a long enough warm-up period has passed to reach a steady state (which, without any intervention, occurs at around day 50), and that the results are reported after the simulation reaches equilibrium. Percent reduction (PR) values in mosquito abundance are calculated by averaging 30-day abundances (after the population reaches a steady-state) from two intervals where applicable: before and after applying the intervention(s) to the base mosquito population. Each simulation is run five times and then the average is reported. The model’s output (FA) is compared against the real-life data. As real data is in the scale of 0–500, the actual output of the model is scaled down by 100 (e.g., if female abundance is 6400 for a day, by scaling it would be 6400/100=64) and then compared to the real-life data. Since the model’s output is generated on a daily basis, a separate calculation is made for calculating the FA for a month. For each month, output is taken to be the average of all days in a month. All simulations are run as a single-threaded program, in a single-process on an Intel machine with Intel (R) Core (TM) i3 CPU having 8GB RAM.

## Results

### Impact of average and composite temperatures

Using the daily average temperature with a generic $40 \times 40$ landscape having a *non-absorbing* boundary (generated by *VectorLand* [29]) did not yield a good fit with the corresponding real-life data (Fig. 3a). Several experiments have been conducted with a number of combinations. In one experiment the maximum temperatures recorded for each day in February and March, and the minimum temperatures recorded for each day in April and May (with the same landscape) have been used. As shown in Fig. 3b, this produced a better fit with the real-life data: the FA magnitudes during February and March match closely, decrease immediately after March, and continue to decrease until June.

### Impact of daily temperature using a generic landscape

The impact of applying daily temperature in the simulation as opposed to a constant temperature throughout was examined using a $40 \times 40$ sized *non-absorbing* landscape generated by *VectorLand* [29]. As is evident from the results presented in Fig. 4a, the abundance of adult female mosquitoes with daily temperature usage is much higher than that with the constant temperature usage. Also the former has a high peak during March but there is no such indication in the latter despite that for some other months, both the output patterns are quite similar.

### Impact of daily rainfall using a generic landscape

Figure 4b illustrates the impact (on the results) of the daily rainfall data usage from a weather profile (referred to as the DR model) for each day of the simulation against no rainfall/constant rainfall data (i.e., 1.0) usage (referred to as the NR model) for the full simulation. From April to December the female abundance of the NR model is much more higher than that of the DR model. Since heavy rainfall results in the loss of larvae, the DR model produces less females.

### Baseline abundance for Bandarban

Following some verification and validation (V and V) experiments, the resulting baseline FA curve as shown in Fig. 5 is considered to be acceptable from a realistic point of view. From the graph it is easily observed that there is a high peak during March. During January and December FA is lower compared to other months. This FA curve is used for comparison when various vector control interventions are applied.

### Interventions

All interventions (ITNs, IRS, and LSM) are applied into ABM$_{vagus}$ in isolation and in combined mode. The FA produced by any experiment is compared with the baseline FA (without any vector control interventions applied). Results of applying interventions are discussed below.

#### ITNs in isolation

To examine the effect of ITNs in isolation, a number of experiments based on different parameters stated in Table 4 were conducted. Figure 5 illustrates the results. Significant decrease of the FA after applying the intervention is clear from the figure. In fact, Fig. 5 shows that the FA decreases drastically as a result of ITNs application with the parameter setting #2 in Table 4 with *household-level complete* coverage. As can be seen, after a certain period, the FA magnitude goes to zero (i.e., 0) for this parameter setting. This result can be attributed to the fact that under this setting, mosquitoes are not able to get any blood meal from any house at all. Recall that, only humans are considered as blood source in our model.

When ITNs are applied with the same parameter setting (i.e., setting #2 in Table 4) but with *household-level partial coverage with multiple chances*, the FA reduction is found to be lower (in comparison to the household-level complete coverage). Note that ITNs with household-level *partial* coverage with multiple chances assumes that 2 persons of a house are protected among all members. Therefore, in this case, mosquitoes have quite a good chance to get blood meal to increase their abundance.

Based on the above experiments it is observed that ITNs with* household-level complete* scheme produces better results compared to the ITNs with* household-level partial* scheme. However, the former is somewhat unrealistic in nature; on the contrary, in the latter, a fraction of persons are not protected resulting in a lower performance.

#### IRS in isolation

When IRS in isolation were applied with the parameter setting #3 in Table 4, FA is decreased, albeit insignificantly (Fig. 6). However, for the parameter setting #4 in Table 4, the FA is decreased significantly; in fact, the FA goes near zero in some cases.

Recall that, IRS is applied in the BMD stage. In the BMD stage, if *R* is set as low, then the mosquitoes are more likely to stay at the households and get killed (due to IRS). Based on the above experiments it is observed that the FA reduction depends on the configuration of IRS parameters: high coverage (i.e., 100%), high mortality rate (i.e., 80%) and low repellency rate (i.e., 10%) seem to produce better results.

#### LSM in isolation

Figure 7 presents the results when LSM in applied in isolation for both parameter settings #5 and #6 in Table 4. As can be seen, for this intervention, the FA reduction is not very significant. However, in both cases, the reduction is consistent throughout the year.

#### ITNs with LSM

Figure 8 illustrates the results of applying ITNs *household-level complete* scheme with LSM in combination with parameter setting #7 in Table 4. The significant reduction in the FA has been noticed and that too quite consistently throughout the year. In fact, for most of the post-intervention period, the FA reduction is around 38% or more compared to the baseline FA.

#### IRS with LSM

Figure 8 illustrates the results of applying IRS with LSM in combination with parameter setting #8 in Table 4. Significant reduction in FA can be noticed after applying IRS and LSM in combination with this setting. In fact, after the interventions are applied, the average FA reduction is approximately 21% compared to the baseline FA, and is consistent throughout the post-intervention period.

#### ITNs with IRS

ITNs with IRS in combination have been applied with parameter setting #9 in Table 4. As can be seen from Fig. 8, very significant reduction in the FA can be noticed for this setting. For most of the post-intervention period, the FA is decreased by more than 42% compared to the baseline FA and the reduction is consistent throughout the year.

From the combined results (as illustrated in Fig. 8) it is evident that ITNs–IRS combination produces better results compared to other combined interventions experimented above. It is also seen that any intervention combined with ITNs produces better results.

#### ITNs, IRS and LSM in combination

Application of ITNs, IRS and LSM interventions in combination using the parameters mentioned in Table 5 provides some interesting results. Figure 9 shows the percent reductions in mosquito abundance as a function of LSM coverage, ITN coverage and IRS coverage when LSM, ITNs and IRS are applied in combination. It has been observed that while IRS coverage increases with LSM and ITNs, FA reduction percentage is increased significantly.

#### Three versus two interventions in combination

ITNs, IRS and LSM have been applied with average protection coverage in different combinations. The result of these combinations are discussed in earlier sections. Figure 9 provides an opportunity to compare FA reduction percentage when three interventions are applied with the case when two different types of interventions are applied in combination. From the figure several things are evident:

- FA reduction percentage is higher in ITNs, IRS and LSM combined mode than other combinations.
- FA reduction percentage of ITNs with LSM is more consistent as compared to others.
- FA reduction percentage of IRS and LSM combination is much more fluctuating and is the lowest among others.

## Discussions

ABM$_{vagus}$ is developed for *An. vagus* based on the field data and the equations drawn in other studies applicable for this model, the environmental factors are incorporated and finally some vector control interventions including IRS, LLINs, and LSM are applied on this model to analyse the impact of these approaches. Some key characteristics, observations and limitations of this study are discussed below.

### The core model

To date, many studies have concentrated on modelling *An. gambiae* because it is one of the most important vector in Africa [99], however, species which are potential for malaria transmission and are under consideration of malaria transmission in the other malaria endemic areas like Bangladesh [8] were not studied well. Therefore, a core model for *An. vagus* is produced. The combinational approaches, i.e., the equation based modelling of some stages and the probability based modelling of some stages make the core model more realistic seen in the result for *An. vagus*. Although this core model has been developed based on the biological attributes of *An. vagus*, it can be seen as a framework for researchers to use or modify it for other similar species as well. In particular, the model is able to show the effects of some environmental or other factors associated with the life cycle of a species which could be a useful feature for researchers working in a relevant area. Some miscellaneous issues, mentioned in Arifin et al. [30] are also applicable for the core model. This is because some equations as well as some approaches of various stages of mosquitoes are taken directly from that study. Some of the benefits of the core model are briefly highlighted below.

#### Generalization of the ABM

The ABM developed through this research work is a form of a generalized technique. This methodology may be applied to similar species by replacing some special attributes of *An. vagus*. For example, in pupal stage it was possible to change the percentages of pupae for a certain period. Another example, we were able to use the zone specific weather data as input for the model. Subsequently, for that zone specific real-life data can be compared with the output of the newly developed model.

#### Seasonal patterns

Incorporation of the daily weather data into this model produces more realistic output for *An. vagus* which is observed in Fig. 3. Unlike other studies [26, 29, 30] in which a fixed temperature is used for the full simulation period, ABM$_{vagus}$ uses the temperature daily in various stages of the life-cycle. As a result, this model generates the seasonal pattern of vector abundance which is more weather specific. Hence, by using this model, it is possible to get an option to set zone specific weather data as input to observer the seasonal pattern of vector abundance for that zone.

#### Impact of rainfall on mortality rates

To incorporate the impact of rainfall on the daily mortality rate of all immature stages the existing mortality rate defined by some studies [26, 29, 30] were modified and introduced a new parameter, the quantifier, $\sigma _s$ additionally to make core model closer to real life.

By varying the quantifier, $\sigma _s$ in Eq. 1, the researchers were able to see the impact of daily mortality rate of any immature stage for other mosquitoes. This approach will help to explore the impact of rainfall and its reflection on vector abundance.

#### Real-life data to generate landscapes

When geographic information system (GIS) data is not available for a particular area, the respective landscapes have been designed and developed using the field data of breeding sources. When GIS data of an area is not available, the above mentioned techniques can be very useful for researchers for generating landscapes for that area using field data.

#### Docking of ABMs

Following the literature [26, 28–30], docking techniques have been used to verify and validate (V and V) the core model. For docking the core model, several different versions of the ABM are generated by changing some configurations/mechanisms in some stages based on the field data or the data found in the literature. Among all different versions of the ABM, ABM$_{vagus}$ produces the output which fits to the real-life data closely.

### Interventions

Following the work of Arifin et al., some interventions (such as, ITNs, LSM) [29] have been incorporated in the model. Additionally, the logic for incorporating IRS as an intervention has been introduced into the model. Some discussions on the applied interventions in different modes as briefly highlighted below.

#### Incorporation of IRS

The IRS logic that has been developed here can be used in other models for other* Anopheles* species. The logic may also be modified as and when appropriate.

#### Applying interventions in combination

It has been observed from Fig. 9 that ITNs with IRS in combination produces better results compared to ITNs with LSM and IRS with LSM in combination. This output is also supported by some studies. Mohammad Shafiul Alam and Hasan Mohammad Al-Amin have said that when ITNs with IRS is applied in combination, mosquitoes would not be able to bite, fed on humans inside and could not rest inside and thus, ITNs with IRS can produce better result compared to others (personal communication, January 15, 2014). MSA and HMA also argued that for IRS with LSM and ITNs with LSM in combination would only provide good results if the vectors oviposit in permanent or semi-permanent habitats which are fixed, findable and few. Hence, if vector oviposition sites are few and findable, LSM would be the best approach. From the field studies (Al-Amin, HM and Alam, MS, personal communication, 2013), mosquito oviposition sites are not fixed nor few and for such situations WHO [100] suggests core (i.e., ITNs and IRS) interventions.

One of the major observations from Fig. 9 is that when ITNs, IRS and LSM are applied in combination it produces better output compared to the output produced by any two interventions together in combination. This is also affirmed by some studies: for example, *An. vagus* mostly rests indoor [101, 102] so the chances of contact with ITNs and IRS are greater for *An. vagus*; if LSM is added in the intervention it will produce even better results for mosquito control because the survived mosquitoes from ITNs and IRS will not find enough oviposition sites to increase its population.

It has been observed that ITNs, IRS, and LSM in combination produces better result than any other two interventions for the Bandarban area. Hence, applying these three interventions altogether (in combination) is recommended whenever possible. It has also been observed that ITNs combined with IRS produces better outcome than any other combination of two interventions for the Bandarban area. Thus, when combination of three interventions (i.e., ITNs, IRS, and LSM) is infeasible, ITNs combined with IRS should be applied.

### Limitations and miscellaneous issues

In this section the limitations of the core model are discussed along with other miscellaneous but important issues. For the development of all aquatic stages, the air temperature has been used instead of water temperature due to the unavailability of the latter. Rainfall data is not considered for increasing aquatic habitats for making the core model simple. Other environmental factors with temperature and rainfall incorporation would make the model to perform better. Also, a number of spatial factors need be incorporated to the model to make it truly a spatial ABM.

As the real-life field data for female abundance of *An. vagus* at Bandarban, the data reported in [8] have been used. The authors of [8] used CDC miniature light trap for trapping mosquitoes. Traps were placed for 12 h (6 p.m. to 6 a.m.). During the wet (dry) season 100 (50) houses were selected randomly. So, presumably, using more traps in more houses could have resulted in a higher abundance value. This in fact prompted us to focus on the pattern of the curve for real-data validation rather than the actual values of the real-life data. During the V and V process major differences between real-life data and the simulated output have been seen during the months of February, March, April and May. As mentioned before, the maximum temperature of each day for February and March and the minimum temperature of each day for April and, May and the average temperature for other months produces output which much more closer to the real-life data. Note that, extensive experiments have been carried out considering different models/configurations/equations at different stages of the life cycle. As a result, a number of versions of the model have been produced. In the sequel, the output of ABM$_{vagus}$ has been found to be much more realistically closer to the real-life data. Arifin et al. [29] has described some miscellaneous issues are also applicable for the core model too since some equations or some approaches of various stages of mosquitoes are taken directly from that study.

As has been mentioned above, several versions of ABM$_{vagus}$ have been implemented in this research work. These versions differ from each other on different parameter combinations as well as on how the biological life cycle has been implemented within the core model. For example, in one version, 12 stages of life cycle have been implemented. However, during validation it failed to match the seasonal pattern. This is why the results of this model is not reported here. The reason for this mismatch may be attributed to a number of simplified assumptions taken during the core model development (e.g., not incorporating rainfall effects on habitats, water temperature etc.) which may have some complex dynamics in this case. Another reason could be the lack of enough real-life data for the sub stages. Further investigation along this line is planned.

Continuing on the discussion of several versions of ABM$_{vagus}$, it is worth-mentioning that experiments have also been conducted with different landscapes considering separate configurations for wet and dry seasons. Unfortunately, when separate configurations for dry/wet seasons were used, the results failed to follow the real-life data pattern. Why did the seasonal configuration fail? The assumptions were probably unrealistic. Also, the real-life female abundance data was a monthly estimate whereas the configuration planned was seasonal; this did not help either. On the contrary, a single configuration had captured an average scenario which provided us a good match with the real-life data pattern. Recall that, in this research, the highest importance has been given to the issue of matching the real-life data pattern for validation purposes. And this is believed to be the first attempt to do that.

Finally, one positive thing of this complete research work lies in the fact that ABM$_{vagus}$ is capable of taking any configuration as input and run simulations to produce results accordingly. Since the intervention logic has been incorporated in the model independent of any such configuration, ABM$_{vagus}$ will definitely give more accurate results and recommendations once the more realistic configurations are given as input. In fact, as a future research, a data acquisition project for mosquito habitats to get a realistic configuration, preferably on a monthly basis, is planed.

## Conclusion

A conceptual model in malaria epidemiology is developed by utilizing the biological attributes relevant to the vector population dynamics of *An. vagus* and its agent-based implementation (i.e., ABM$_{vagus}$) is described. Although ABM$_{vagus}$ is developed specifically for *An. vagus* of a particular area, it is a form of a generalized technique; hence it may be applied to similar species of *An. vagus*.

After incorporating environmental factors the model is able to produce the seasonal patterns of *An. vagus* abundance. It is also observed that the pattern of *An. vagus* abundance based on the environmental factors is quite different than that obtained using fixed temperature throughout the simulation period. Notably, the output of ABM$_{vagus}$ is highly dependent on real-life field data of the environmental factors and the landscape of a particular area. Hence, it is recommended to use ABM$_{vagus}$ for a given area with its local field data.

During the V and V process, it was observed that DMR of larvae and the value of CC assigned into the aquatic sources play important roles in the vector population dynamics. However, the exact locations and patterns of households situated in the artificial landscape had less impact on vector abundance.

For high malaria endemic areas like Bandarban, applying all vector control interventions (ITNs, IRS, and LSM) in combination is recommended. The simulations also demonstrate that ITNs with IRS can be applied when all three interventions in combination are not feasible, and that ITNs with LSM are less likely to produce consistent reduction in abundance.

In future, additional features such as the malaria parasite life-cycle, incorporation of GIS data and other weather parameters (e.g., humidity, cloudiness, desiccation), alternative hosts for blood-feeding (e.g., cattle), additional fine-tuned biological attributes of *An. vagus* (e.g., vector resistance) and variation of its flight range could be planned to include in the model. Adoption to the changes of breeding sources due to rainfall is another direction for future research. Lastly, considering the mix of vector species in endemic regions like Bandarban, a more realistic model capable of handling multiple species within a single ABM could also be studied to best address malaria transmission.

## Additional files

**Additional file 1.** Brief description of some model presented in [26, 29] Model of Larval Stage.

**Additional file 2.** ABMvagus (in ZIP format).

**Additional file 3.** Sample input file (in XML format) for the ABMvagus.

**Additional file 4.** Sample weather data input file (in CSV format) for the ABMvagus.

## Abbreviations

ABM: agent-based model
AH: aquatic habitat
BMD: blood meal digesting
BMS: blood meal seeking
C: coverage
CC: carrying capacity
CDC: Centers for Disease Control
CHTs: Chittagong Hill Tracts
DMR: daily mortality rate
E: egg
FA: female abundance
G: gravid
HH: human habitat
IA: immature adult
IRS: indoor residual spraying
ITN: insecticide-treated net
L: larva
LLIN: long-lasting insecticidal net
LSM: larval source management
M: mortality
P: pupa
R: repellence
VA: vector abundance
V and V: verification and validation
WHO: World Health Organization
