# Supplementary material for: A spatial agent-based model of Anopheles vagus for malaria epidemiology: examining the impact of vector control interventions
Source: Malar J. 2017 Oct 27;16:432. doi: 10.1186/s12936-017-2075-6 (PMC5658966; doi:10.1186/s12936-017-2075-6)
Supplement: Supplementary file 1 — Additional file 1. Brief description of some model presented in [26, 29] Model of Larval Stage. [file 12936_2017_2075_MOESM1_ESM.pdf]

Additional file 1: Brief description of some model presented in [26,29]

Model of Larval Stage

A temperature driven framework for the larval development is described in some previous studies [26,29,30]. Based on that the larval development rate per hour at temperature $T(^oK)$ is given in Equation 9.

$$LarvalDevelopment_{perhour}(T) = \frac{\rho_{25^oC} \cdot \frac{T}{298} \cdot \exp(\frac{\Delta H_A^{\neq}}{R}(\frac{1}{298} - \frac{1}{T}))}{1 + \exp(\frac{\Delta H_L}{R}(\frac{1}{T_{\frac{1}{2}L}}) - \frac{1}{T}) + \exp(\frac{\Delta H_H}{R}(\frac{1}{T_{\frac{1}{2}H}}) - \frac{1}{T})} \quad (9)$$

Here $\rho_{25^oC} = 0.00415, \Delta H_A^{\neq} = 15684, \Delta H_L = -229902, T_{\frac{1}{2}L} = 286.4, \Delta H_H = 822285, T_{\frac{1}{2}H} = 310.3$ and R=1.987.

Since Equation 9 is on hour time unit for a given day (e.g. the ith day), so the daily larval development rate can be found in Equation 10 below.

$$LarvalDevelopment_{perday}(i) = \sum_{h=0}^{24} LarvalDevelopment_{perhour}(T_i) \quad (10)$$

Here T_i is the temperature for i^{th} day.

By putting all above mentioned values into Equation 9, we can get the following Equation 11 for larva development per day.

$$LarvalDevelopment_{perday}(i) = (T_i \times 0.000305 - 0.003285) \times 24 \quad (11)$$

The threshold for larval development, *LarvalDevelopmentThreshold*, represents the threshold for the larval stage (i.e., when the development is complete, and the pupation begins). To allow 10% variability, it is chosen from a normal random variable G, with *mean* 1.0 and *standard deviation* 0.1:

$$LarvalDevelopmentThreshold = 1 + G(0, 0.1) \quad (12)$$

And the cumulative development of larvae after x days can be shown using Equation 12 below.

$$CumulativeLarvalDevelopment = \sum_{i=1}^{x} \sum_{h=0}^{24} LarvalDevelopment_{perhour}(T_i) \quad (13)$$

Each day, each larva approaches its threshold by developing at a rate characterized by Equation 11, and $LarvalDevelopment_{perday}(i)$ is accumulated into the cumulative larval development time, *CumulativeLarvalDevelopment*. The larva transforms to a pupa when the following condition is satisfied:

$$CumulativeLarvalDevelopment \geq LarvalDevelopmentThreshold \quad (14)$$

Model of Immature Adult Stage

Generally when a mosquito emerges from water, it stays in the *Immature Adult (IA)* stage for one to three days, depending on temperature [30]. Arifin *et* al. [30] has described a function shown in Equation 15 for modelling IA stage. According to this equation, when the temperature is at 36°C, 27°C, and 18°C, the corresponding development time in the *IA* stage is 1, 2, and 3 days, respectively.

$$ImmatureAdultDevelopment_i(T_i) = -2.67 \times T_i + 120 \qquad 15 \leq T_i \leq 40 \quad (15)$$

Model of Mate Seeking Stage

Arifin *et* al. [30] assumed the mate seeking stage instantaneous. It describes that mate seeking occurs during the first hour of evening (i.e. before 19.00).

Model of Blood Meal Digesting Stage

As per Arifin *et* al. [30] the *BMD* stage is a highly temperature dependent stage, where mosquitoes at different temperatures take 1∼2.5 days to digest blood meals and develop their eggs. The linear function that has been developed by Arifin *et* al. [30] describes their relationship as presented in Equation 16

$$BloodmealDigesting_i(T_i) = -1.23 \times T_i + 77 \qquad 15 \leq T_i \leq 40 \qquad (16)$$

Here i is a day and T_i is the daily temperature for the ith day.

Model of Gravid Stage

Generally in the *Gravid* stage, a female mosquito lays a clutch of developed eggs. Arifin *et al.* 2014 [30] assumes that a female in the G stage has a 25% probability to randomly sample a habitat at each time unit during the 12-hour time window (from 6.00PM to the following 6.00AM) every day. If all eggs are not laid within one night (i.e., the 12-hour time window mentioned above), the mosquito retains her eggs, finds a place to rest still staying in the G stage, and waits for another night to lay the remaining eggs. After laying all her eggs, and the time-window (from 6.00PM to the following 6.00AM) permits, it enters the BMS stage, thus starting a new gonotrophic cycle.

Mortality in the adult stages

In [26,30] the authors have presented modified version of the logistic mortality model in which the age-dependent component of mortality increases exponentially with age. When an adult emerges, it starts with a daily mortality rate of α. As the age of the mosquito increases, the age-specific mortality rate $ASMR_{Age(adults)}$ for that age-cohort changes based on the following equation:

$$ASMR_{Age(adults)} = \frac{\alpha \times e^{\frac{Age}{\beta}}}{1 + \alpha \times s \times \beta(e^{\frac{Age}{\beta}} - 1)} \qquad (17)$$

Here, α is the baseline daily mortality rate, β is the inverse of exponential mortality increase with age, s is the degree of mortality deceleration, and Age is the common age of the age-cohort. The number of adults that are removed from the system, $ToKill_{Age(adults)}$, is then computed as follows:

$$ToKill_{Age(adults)} = ASMR_{Age(adults)} \times Adults_{Age} \tag{18}$$

Here, $Adults_{Age}$ is the size of the adult age-cohort.
