# Supplementary material for: A spatial agent-based model of Anopheles vagus for malaria epidemiology: examining the impact of vector control interventions
Source: Malar J. 2017 Oct 27;16:432. doi: 10.1186/s12936-017-2075-6 (PMC5658966; doi:10.1186/s12936-017-2075-6)
Supplement: Supplementary file 2 — Additional file 2. ABMvagus (in ZIP format). [file 12936_2017_2075_MOESM2_ESM.pdf]

Additional file 2: ABM$_{vagus}$ (in ZIP format).

The ABM$_{vagus}$ is developed in Java (Version: 1.8), using the Eclipse SDK (Version: Mars.2 Release (4.5.2)), which is freely available from [101]. The computer must have the Java Runtime Environment (Version: 1.8 or upper which can be downloaded from [102]) to run the JAR file. Instructions: (1) Download the ZIP file from `https://github.com/zahangirbd/ABMvagus` and extract it into a directory; (2) navigate into the extracted directory: for Windows, use chdir or cd in a command prompt; for UNIX/Mac, use cd in a terminal; (3) execute the command: java -Xms1g -Xmx8g -d64 -jar ABMvagus.jar StandardLandscape.xml WeatherData.csv optionalId where optionalId refers to an optional integer (e.g., 123) to sequence different simulation runs. To change simulation parameters, modify the sample (e.g., StandardLandscape.xml) input file. The runtime generally ranges a few hours. The ABM$_{vagus}$ will create two folders and some plain-text files inside each folder. Each folder or filename is created based on the major parameters (as extracted from the input XML file). Output files are (1) the *.FA.txt file, which contains the number of female adult mosquitoes in the system (one entry per day), and (2) the *.xml file, which contains information about the simulation job, including a listing of the major parameters and simulation events (e.g., application of interventions). Multiple/batch runs can be executed for the same input XML file by using simple scripts (e.g., UNIX shell scripts).
