# Supplementary material for: A spatial agent-based model of Anopheles vagus for malaria epidemiology: examining the impact of vector control interventions
Source: Malar J. 2017 Oct 27;16:432. doi: 10.1186/s12936-017-2075-6 (PMC5658966; doi:10.1186/s12936-017-2075-6)
Supplement: Supplementary file 3 — Additional file 3. Sample input file (in XML format) for the ABMvagus. [file 12936_2017_2075_MOESM3_ESM.pdf]

## Additional file 3: Sample input file (in XML format) for the ABM$_{vagus}$

The ABM$_{vagus}$ (Additional file 2) requires an XML file as its one input. The standard text editor (e.g., Notepad for Windows, TextEdit for Mac, etc.) can be used to view and to modify. It contains various parameters of the model, the simulation to be run, the landscape to be used, and the interventions to be applied. Model parameters include length of the simulation (maximum time-step), initial number of female adult mosquitoes, fecundity (mean and standard deviation), daily mortality rates for eggs and pupae, daily baseline mortality rates for larvae and adult stages of the mosquito life cycle and rainfall quantifier for daily mortality rates. Landscape parameters are width and height of the landscape, the boundary type to be used (Absorbing or Nonabsorbing), and maximum number of moves allowed for a mosquito agent per day. Simulation parameters includes all aquatic habitats and blood meal locations (houses) as individual sub-environments. Each sub-environment parameters includes an optional identifier, its spatial location (x- and y-coordinates), carrying capacity (for an aquatic habitat), number of persons (for a blood meal location), and whether the sub-environment is covered by LSM or ITNs or IRSs. Intervention parameters include the beginning time-step to apply the intervention, coverage, and repellence, mortality/insecticidal effect (for ITNs and IRSs). The current model supports LSM, ITNs and IRSs as interventions. This sample file specifies 258 aquatic habitats, 113 houses, 30% LSM coverage, 50% ITN coverage, 50% IRS coverage, no repellence, 50% ITN mortality and 50% IRS mortality (insecticidal effect).
