# Supplementary material for: A spatial agent-based model of Anopheles vagus for malaria epidemiology: examining the impact of vector control interventions
Source: Malar J. 2017 Oct 27;16:432. doi: 10.1186/s12936-017-2075-6 (PMC5658966; doi:10.1186/s12936-017-2075-6)
Supplement: Supplementary file 4 — Additional file 4. Sample weather data input file (in CSV format) for the ABMvagus. [file 12936_2017_2075_MOESM4_ESM.pdf]

Additional file 4: Sample weather data input file (in CSV format) for the ABM_{vagus}

The ABM_{vagus} (Additional file 2) also requires an weather data file as its another input. The standard text editor (e.g., Notepad for Windows, TextEdit for Mac, etc.) can be used to view and to modify. It contains four columns. They are Date, Max (i.e., Maximum temperature of the day), Min (i.e., Minimum temperature of the day), Avg(i.e., Average temperature of the day), Rainfall (in mm of the day).
